# In silico design of a matrix (M) protein–based vaccine candidate against Newcastle disease virus

**DOI:** 10.1016/j.psj.2025.106101

**Published:** 2025-11-11

**Authors:** Zainab Kamran, Maaz Waseem, Muhammad Asghar

**Affiliations:** a Atta-Ur-Rahman School of Applied Biosciences (ASAB), National University of Science and Technology (NUST), Islamabad, Pakistan; b School of Mechanical and Manufacturing Engineering (SMME), National University of Science and Technology (NUST), Islamabad, Pakistan; c Biology Department, Lund University, Sweden; d Department Sports Sciences and Clinical Biomechanics, University of Southern Denmark, Denmark

**Keywords:** NDV, *In-silico* vaccine, Molecular docking, Computational analysis, M-protein

## Abstract

Poultry industry worldwide: Newcastle Disease (ND) is highly contagious poultry viral disease posed a major threat to the poultry industry due to millions of deaths worldwide. Newcastle Disease Virus (NDV) cause recurrent epidemics due to its rapid genomic evolution. In this study, we developed an *in silico* vaccine against all NDV strains using matrix protein. A reverse vaccinology approach was employed to develop the vaccine. B- and T- cell epitopes that were antigenic, virulent, non-allergenic and MHC alleles binders were selected and linked via linkers. To enhance the immunogenicity EAAAK adjuvant has been linked with N-terminal of the vaccine. The 3D structure of the vaccine was constructed, refined, and validated. The vaccine showed high potential for immunogenicity, non-allergenicity, and antigenicity. Molecular docking confirmed strong binding to chicken Toll-like receptor-4 (TLR4), and subsequently, the *in-silico* immunological simulations depicted a robust and sustained immune response to the vaccine. Computational analyses suggested that the vaccine is physically stable and highly immunogenic, making it a promising candidate against NDV. This study highlights the efficacy of the vaccine design, offering a promising solution for NDV outbreaks in Pakistan and similar regions at the time of emerging infectious diseases.

## Introduction

Newcastle Disease (ND) is a contagious avian disease affecting various domestic species. ND is caused by Newcastle Virus (NDV) is highly contagious, causing severe respiratory, digestive, and neurological issues in birds, as well as detrimental effects on birds. NDV belongs to the *Paramyxoviridae* family also known as avian paramyxovirus type 1 (APMV-1), a single-stranded, negative-sense, non-segmented, enveloped RNA virus (approximately 15 kb) with a broad host range, capable of infecting over 240 species of birds (Maqbool et al., 2023). NDV predominantly affects birds, particularly poultry such as chickens and turkeys (Alexander et al., 2004).

NDV has various strains that are broadly categorized into three main pathotypes: lentogenic (mild), mesogenic (moderately virulent), and velogenic (highly virulent) (Tavassoli et al., 2024). Classical vaccines have been developed against NDV in countries where it is mainly endemic. Inactivated or live vaccines are created using low-virulence strains, but they have become ineffective due to the virus's rapid evolution. Since vaccination is essential, large-scale, regular immunization programs are crucial to protect poultry populations. However, there are several challenges in developing effective vaccines, such as hypersensitivity, insufficient attenuation, longer development times, high costs, and lower immunogenicity (Bambini and Rappuoli, 2009). Therefore, there is a pressing need for novel vaccine candidates that provide broader and more durable protection against genetically diverse NDV strains.

Immunoinformatics and reverse vaccinology approaches have revolutionized antigen discovery by identifying conserved epitopes computationally (Mora et al., 2006). The main strategy for identifying vaccine targets involves finding sequences likely to bind to MHC class I or II proteins for antigen presentation within the pathogen. However, there has been limited research published on NDV in this context (Badawi et al., 2016; Osman et al., 2016). This study specifically targets the NDV matrix (M) protein to achieve prolonged immunity. The NDV matrix (M) protein represents a promising target due to its highly conserved nature and critical role in virion assembly, budding, and host immune modulation (Pantua et al., 2006). Unlike surface glycoproteins F and HN, which are prone to antigenic variation, the M protein maintains sequence stability across NDV genotypes (Badawi et al., 2016). Furthermore, immunoinformatic analyses suggest that M harbors immunodominant epitopes capable of eliciting T- and B-cell responses (Hosseini et al., 2021; Mozafari et al., 2022). This research note presents an in silico design of a domain-based M protein vaccine candidate for NDV using multi-epitope prediction and computational immune modeling.

## Methodology

### Genome sequence retrieval and proteome data comparative analysis

The complete NDV genome sequences (*n* = 303) were retrieved from the NCBI database. The M protein sequences were aligned using Clustal Omega to identify conserved regions. The quality of the selected protein sequence was assessed using an online tool, Check M. The genomes were then uploaded on Roary to identify the core genome.

### Determination of protein antigenicity and allergenicity

An online server called VaxiJen checked the antigenicity of the protein sequences. The M Protein was identified as a potential antigenic protein by exploiting the Vaxijen v2.0 server (applying a threshold value of 0.5). The epitope was tested for allergenicity through Allertop v2.

### T-cell epitopes prediction

MHC I & II Binding Epitopes were predicted using Nettepi 1.0 and their affinities were predicted using the tool NETmhcCONS.

**MHC Class I.** The epitope of T lymphocytes was predicted using IEDB. Following are the MHC class I epitopes that were analyzed and selected for this study: HLA-B 40:6, 41:4,41:3. As a result, the MHC I with a strong binding value (SB) of 0.5 was finally selected.

**MHC Class II**. The NN-align tool was utilized to assess the MHC-II binding prediction using human allele reference set method 2.22. MHC II epitopes shortlisted for study are DRB1_1481, DRB1_1366, DRB1_1445. The MHC II epitope with an SB value of 0.9 was selected for the study.

### B-cell epitopes prediction

To identify the most suitable antigen that provides humoral immunity, B-cell epitope prediction was carried out by ABC pred. Protein amino acid sequence was provided, with a threshold of 0.5. The window length for epitope prediction was chosen as default. The B-cells with a score of 0.8> were selected and the epitopes with an antigenicity score of 0.6> were predicted using Allertop v2.

### Domain discovery and vaccine construction

The tool BLASTp was used to blast both T and B cells to get starting and ending points. Following blast, the sequences were subjected to filters through InterPro. Vaxijen 3.0 system and Allertop v2 were used to predict the allergenicity and antigenicity of vaccine construct.

### Characterization of prioritized vaccine candidates

Some of the physiochemical properties of the proteins like the Instability index, Aliphatic index, and grand average of hydropathicity (GRAVY) were evaluated through Protparam at Expasy (https://web.expasy.org/protparam/protparam-doc.html). The solubility of all the vaccine candidates was predicted by SOLpro It analyzed the vaccine candidates' solubility by including the proteins holding a default probability of 0.45 and above.

### Molecular docking

Immune receptors, found in immune cells, are vital in triggering an immunological response. Molecular docking checks the interaction between a ligand and a protein. The Toll-like receptors TLR4 and TLR5 of chicken were employed for this study and subsequent molecular docking was performed by HDOCK. The interacting residues between the docked complexes were mapped with the help of PDBsum. The structures were refined and tested for stability and flexibility using molecular dynamics (MD) simulation studies. The GROMACS program version 2021.2 was utilized for the molecular dynamics of constructed vaccine.

### Codon optimization and in silico cloning

Codon optimization of the vaccine constructs was performed using the web server, GENEius (available at: https://www.geneius.de/GENEius/Security_login.action). The constructed vaccine was codon-optimized to improve *E.Coli*'s bacterial expression, and the optimized codons were then inserted into the vector pET28a(+) employing the SnapGene® software (from Dotmatics; available at snapgene.com).

### In silico immune simulation

The final constructed vaccine was subjected to a web server, called C-ImmSimm to analyze the kind of immune response generated (available at: http://kraken.iac.rm.cnr.it/ C-IMMSIM/). Default parameters were set on Day 1 and Day 28. Nevertheless, it gave a chance to test the immunogenicity of a generic protein sequence through its amino acid sequence.

## Results and discussion

### Identification of conserved regions and epitope prediction

Multiple sequence alignment of the NDV M protein revealed highly conserved domains across all analyzed isolates, confirming its suitability as a universal vaccine target. Conserved epitopes predicted to bind MHC I and II alleles demonstrated high antigenicity scores (>0.7) and non-allergenic potential. The high conservancy rate (>95 %) among circulating NDV genotypes supports the feasibility of a cross-protective immune response.

### Construction and characterization of the vaccine candidate

The final construct incorporated six T-cell and four B-cell epitopes linked via EAAAK linkers and an adjuvant sequence at the N-terminal (Table 1). The predicted molecular weight (42.3 kDa) and theoretical pI (6.8) suggest a stable, soluble construct suitable for recombinant expression. Structural modeling indicated a compact tertiary structure with favorable Ramachandran plot statistics (>90 % residues in favored regions), confirming the construct’s structural reliability. The designed construct represents a computationally predicted subunit vaccine candidate. While the current work does not experimentally formulate the vaccine, it provides a sequence blueprint for recombinant protein expression and potential subunit vaccine development.

**Table 1:** selected T-cell and B-cell epitopes.

**Selected T-cell Epitopes**							
**Rank**	**Alleles**		**Peptide**		**Binding Energy**		**Binding Level**
123	HLA-B41:04		TERMVFSVVQAPQVL		0.3		<=SB
123	HLA-B41:03		TERMVFSVVQAPQVL		0.5		<=SB
33	DRB1_1481		GKKQIAPQYRIQRLD		0.45		<=SB
34	DRB1_1481		KKQIAPQYRIQRLDS		0.29		<=SB
208	DRB1_1481		VTIDVEVDPKSPLVK		0.32		<=SB
209	DRB1_1481		TIDVEVDPKSPLVKS		0.21		<=SB
174	DRB1_1445		NFVSLTVVPRKDVYK		0.6		<=SB
175	DRB1_1445		FVSLTVVPRKDVYKI		0.71		<=SB
208	DRB1_1445		VTIDVEVDPKSPLVK		0.73		<=SB
209	DRB1_1445		TIDVEVDPKSPLVKS		0.55		<=SB
**Selected B-cell Epitopes**							
**Rank**		**Sequence**		**Score 0.8>**		**Antigencity 0.6>**	
1		FSSSGTACYPIANASP		0.92		**0.7347** (Probable **ANTIGEN** ).	
2		TSTKLEKGHTIAKYNP		0.9		**0.7556** (Probable **ANTIGEN** ).	
2		GKKQIAPQYRIQRLDS		0.9		**0.6565** (Probable **ANTIGEN** ).	
3		PEKIPGSGTLEYKVNF		0.89		**0.7944** (Probable **ANTIGEN**	
4		QVGNEEATVGMINDNP		0.88		**0.9009** (Probable **ANTIGEN** ).	
5		IGLMSTVDKKGKKVTF		0.86		**1.2840** (Probable **ANTIGEN**	
5		AFPIVLQDTGDGKKQI		0.86		**0.7311** (Probable **ANTIGEN** ).	

### Docking and immune interaction analysis

Docking simulations revealed strong interactions between the vaccine construct and TLR4/TLR5 immune receptors, with binding energies comparable to known peptide immunogens. Hydrogen bond analysis indicated stable interactions, suggesting that the construct could activate innate immunity pathways. Similar approaches in other avian pathogens have demonstrated that such docking results correlate with experimental immunogenicity (Osman et al., 2016).

### Immune simulation results

**Fig. 1:**
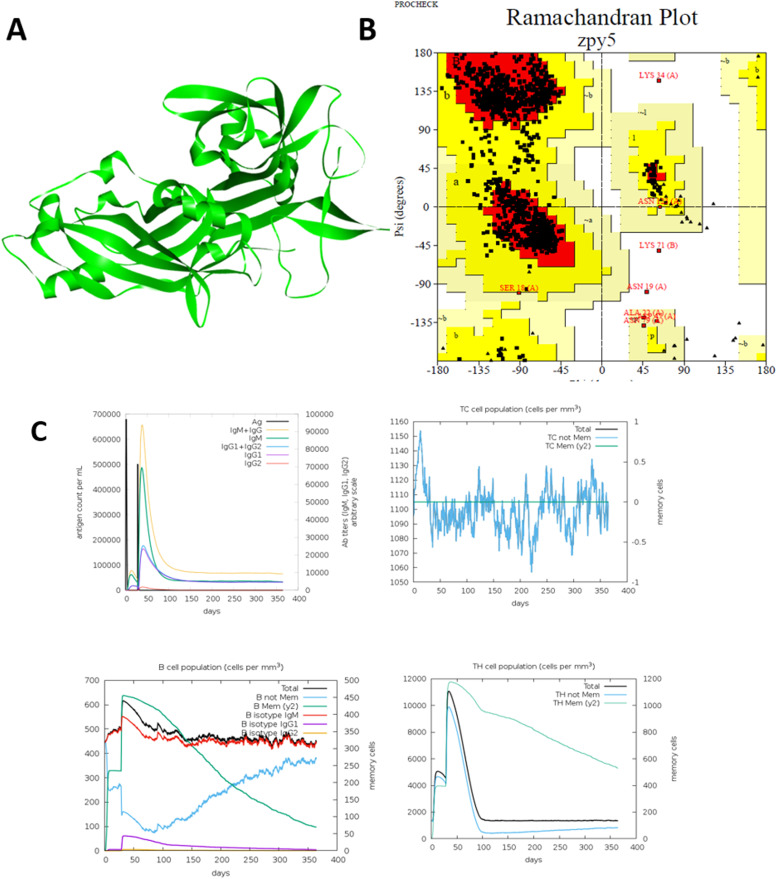
A) Tertiary structure of the constructed vaccine. B) Ramachandran plot for validation. C) In silico immune simulation showing B-cell population, T-cell population, antigen count, and TH-cell population.

Immune simulation using C-ImmSim predicted significant elevations in B-cell and T-cell populations after primary and secondary exposures, along with increased immunoglobulin titers and IFN-γ production. The simulation suggested activation of memory B and T cells, indicating potential for long-term immunity (Fig. 1C). These computational findings align with prior evidence that domain-based multi-epitope vaccines can sustain prolonged immunological memory (Raza et al., 2022).

### Rationale for M protein as a vaccine target

Although F and HN glycoproteins are traditional NDV vaccine targets, they exhibit high genetic variability, often resulting in vaccine escape mutants. In contrast, the M protein’s conserved nature minimizes antigenic drift, providing an opportunity for broad-spectrum and long-lasting protection. Studies by Badawi et al. and Hosseini et al. demonstrated the immunogenic potential of M protein epitopes in stimulating CTL responses (Badawi et al., 2016; Hosseini et al., 2021). Mozafari et al. further supported its use as a complementary target in subunit vaccine design. These computational and immunological analyses have highlighted the NDV M protein’s conserved immunogenic domains capable of eliciting both B- and T-cell. They also validate M as a potential subunit vaccine candidate due to its essential role in viral assembly and relative antigenic stability. The present in silico findings in this study strengthen the rationale for considering M protein-based constructs as viable vaccine candidates for NDV.

### Limitations and future perspectives

While this computational study presents a validated structural and immunological framework for an NDV M protein vaccine candidate, laboratory-based validation is required to confirm the predicted antigenicity, expression stability, and immunogenicity. Future work should focus on expressing the construct in a bacterial or yeast expression system and evaluating immune responses in chicken models. Delivery strategies such as recombinant protein formulation with adjuvants or viral vector platforms can be explored to optimize immune stimulation.

This in silico analysis provides a cost-effective foundation for vaccine development, particularly valuable for endemic regions like South Asia, where ND outbreaks are frequent. The integration of computational prediction and structural modeling allows for rapid prioritization of potential vaccine targets, reducing dependence on costly empirical screening.

## Fundings

National University of Sciences and Technology Pakistan, Lund University Sweden and Ragnar Soderberg Foundation, Sweden.

## CRediT authorship contribution statement

**Zainab Kamran:** Writing – review & editing, Writing – original draft, Visualization, Validation, Methodology, Investigation, Formal analysis, Data curation. **Maaz Waseem:** Writing – review & editing, Writing – original draft, Visualization, Validation, Methodology, Formal analysis, Data curation. **Muhammad Asghar:** Writing – review & editing, Writing – original draft, Supervision, Methodology, Conceptualization.

## Disclosures

The authors declare that they have no known competing financial interests or personal relationships that could have appeared to influence the work reported in this paper.
